# Characteristics and risk differences of different tumor size on localized prostate cancer: A retrospective cohort study in the SEER database

**DOI:** 10.1002/cam4.3856

**Published:** 2021-03-16

**Authors:** Zhen Zhou, Feng Yue, Liang Jin, Xiang Liu, Ting‐Shuai Zhai, Jia‐Xin Zhang, Wen‐Yu Gu, Sheng‐Hua Liu, Ming Luo, Bo Peng, Xu‐Dong Yao, Lin Ye

**Affiliations:** ^1^ Department of Urology Shanghai Tenth People's Hospital，Tongji University School of Medicine Shanghai China; ^2^ Department of Urology，First Clinical Medical College Nanjing Medical University Nanjing China; ^3^ Department of Urology Dalian Friendship Hospital Dalian Liaoning China; ^4^ Department of Urology Heidelberg University Hospital Heidelberg Baden‐Württemberg Germany

**Keywords:** diseases stages, localized prostate cancer, odds radio, prognosis, tumor size

## Abstract

**Objective:**

We aimed to evaluate the role of tumor size in predicting tumor risk for localized prostate cancer (PCa) patients undergoing radical prostatectomy (RP).

**Methods:**

Twenty‐five thousand, one hundred twenty‐seven men with PCa receiving RP from 2010 to 2015 were extracted from the Surveillance, Epidemiology, and End Results database. Kaplan–Meier plots and multivariable Cox regression analyses were used to illustrate overall survival (OS) according to the tumor size. The tumor size was confirmed by postoperative pathology after RP.

**Results:**

Among overall localized PCa, 84.6% were high‐risk PCa, 9.2% were intermediate‐risk PCa, and 6.2% were low‐risk PCa. Multivariate analyses demonstrated that tumor size ≥21 mm was an independent risk predict factor of low‐risk PCa (odds ratio [OR]: 11.940; 95% CI, 9.404–15.161; *p* < 0.001) and intermediate‐risk PCa (OR: 1.887; 95% CI, 1.586–2.245; *p* < 0.001). Tumor sizes ≤5 mm significantly correlated with high‐risk PCa (*p* < 0.001). Tumor size ≤5 mm had the worst OS in overall localized PCa and high‐risk PCa (*p* < 0.001).

**Conclusions:**

In localized PCa, tumor sizes ≥21 mm may help predict low or intermediate‐risk PCa, while tumor sizes ≤5 mm might help predict high‐risk PCa. In clinical practice, we should be on high alert for patients with tumors size ≤5 mm due to its poor prognosis after RP.

## Graphical abstract

We quantified the relationship between tumor size and localized prostate cancer grade, and hoped to play a positive role in early diagnosis and treatment of clinical work.

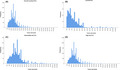

## 1 BACKGROUND

Prostate cancer (PCa) is a serious disease that is harmful to men's health worldwide, ranking first in cancer incidence and second in cancer mortality for males in the United States.^1^ In the United States, more than 160,000 new cases annually are diagnosed as PCa, which accounts for approximately 19% of all new cancer cases, and the lifetime risk of PCa is estimated at about one in six.^1^ Although the incidence of PCa is high, the mortality of PCa is very low. Approximately 8% of all deaths due to PCa among men in the United States.^1^

An increase in survival rates has been noted in recent years due to the extensive use of prostate‐specific antigen (PSA) testing, resulting in a more favorable stage distribution.^2^ The European Association of Urology Guidelines 2019 indicated that localized PCa can be classified into three disease stages: low‐risk, intermediate‐risk, and high‐risk PCa.^3^ The PSA's introduction decreased PCa mortality for decades while brought overdiagnosis concerns. And new biomarkers such as multiparametric imaging are needed to ease these concerns. Different therapy regimens are available based on the clinical stage and individual patients’ circumstances, which include estimated life expectancy, as well as personal values and preferences.^4^ Therefore, it was necessary to figure out the potential factors relatively simple and effective to predict different risks of PCa.

It is commonly considered that large tumor has a poorer prognosis than small tumor and existing studies have shown that tumor size larger than 10 mm might be more aggressive.^5^ But the role of tumor size is still uncertain for localized PCa. Besides, the application of multiparameter magnetic resonance imaging (MRI) in the diagnosis, staging, and treatment of PCa has attracted more and more attention. At present, multiparameter MRI is widely used due to its ultra‐high performance in discrimination, calibration, and clinical usefulness.^6^ The latest research suggested that shorter MRI provides quicker, simpler, and less costly MRI protocols without compromising its effectiveness.^7^ Moreover, the predictive role of multiparameter MRI in intermediate‐risk PCa has been reported.^8^ Therefore, can we look for index lesions in MRI, such as tumor size, and explore its relationship with disease stratification? In this study, we could not directly obtain the index lesion of MRI and the pathological findings of tumor size were used instead.

Consequently, in this study, we investigated the relationship between PCa risk and tumor size in localized diseases to figure out if tumor size could serve as a biomarker for aggressive PCa, thus helping clinical decision‐making.

## 2 METHODS

### 2.1 Patient selection

**FIGURE 1:**
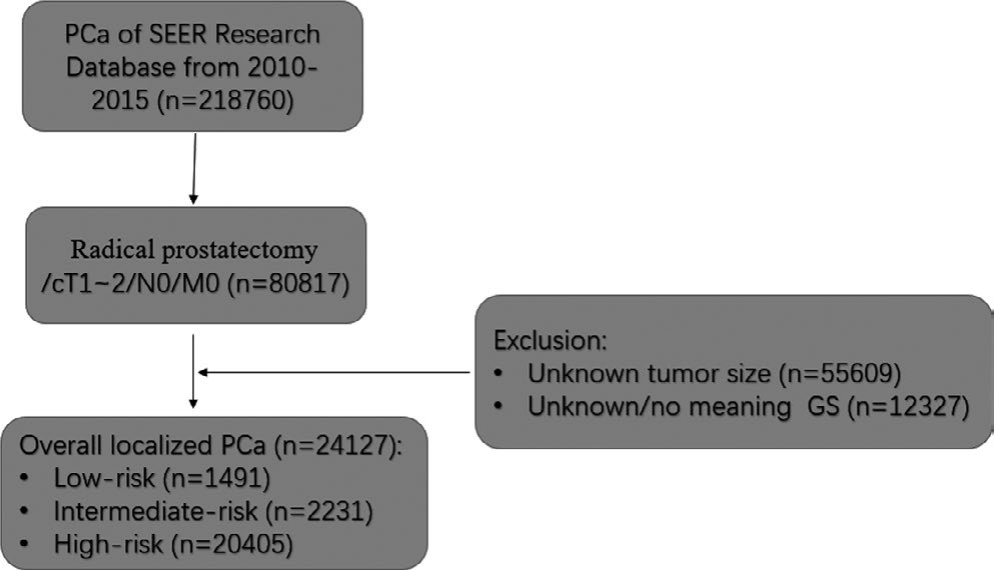
The flow chart describes the steps taken to identify 24,127 localised prostate cancer patients in the Surveillance, Epidemiology, and End Results (SEER) database

Prostate cancer patients from 2010 to 2015 were selected from the Surveillance, Epidemiology, and End Results (SEER) database using the SEER*Stat software program (version 8.3.7). Twenty‐four thousand, one hundred twenty‐seven patients were extracted in this study from the SEER database. All patients were operated with radical prostatectomy (RP). Besides, all of them were diagnosed as clinical T1‐2, N0, and M0. We deleted patients with unknown or meaningless CS tumor size (codes 990–995, 999), age at diagnosis <18, or unknown PSA (codes 988,998,999) (Figure 1).

### 2.2 Definition of variables for analyses

Patients were stratified according to the tumor size. The tumor size was confirmed by postoperative pathology. If PCa is multifocal, the database recorded the size of the largest tumor. Covariates consisted of years of diagnosis, age at diagnosis, race, grade, laterality, PSA, and derived AJCC TNM stage (7th edition, 2010–2015). Low‐risk PCa is defined as PSA<10 ng/ml, and Gleason score (GS)<7, and cT1‐2a, intermediate‐risk PCa is defined as PSA 10–20 ng/ml, or GS 7, or cT2b, and high‐risk localized PCa is defined as PSA>20 ng/ml, or GS>7, or cT2c.

### 2.3 Statistical analysis

We utilized SPSS v25.0 (SPSS Inc.). The *χ* ^2^ test was used to compare clinical characteristics between different groups. Logistic regression analysis was used to analyze the effects of different tumor sizes on the prediction of low‐risk PCa, intermediate‐risk PCa, and high‐risk PCa. The *p* value was set at 0.05. We used multivariate Cox regression analysis to determine the association with overall survival (OS) rate. For data values that were statistically significant, the hazards ratio (HR), odds ratio (OR), and the 95% confidence interval (95% CI) were also generated.

## 3 RESULTS

### 3.1 Identification of tumor size selection to predict the localized PCa stage

**FIGURE 2:**
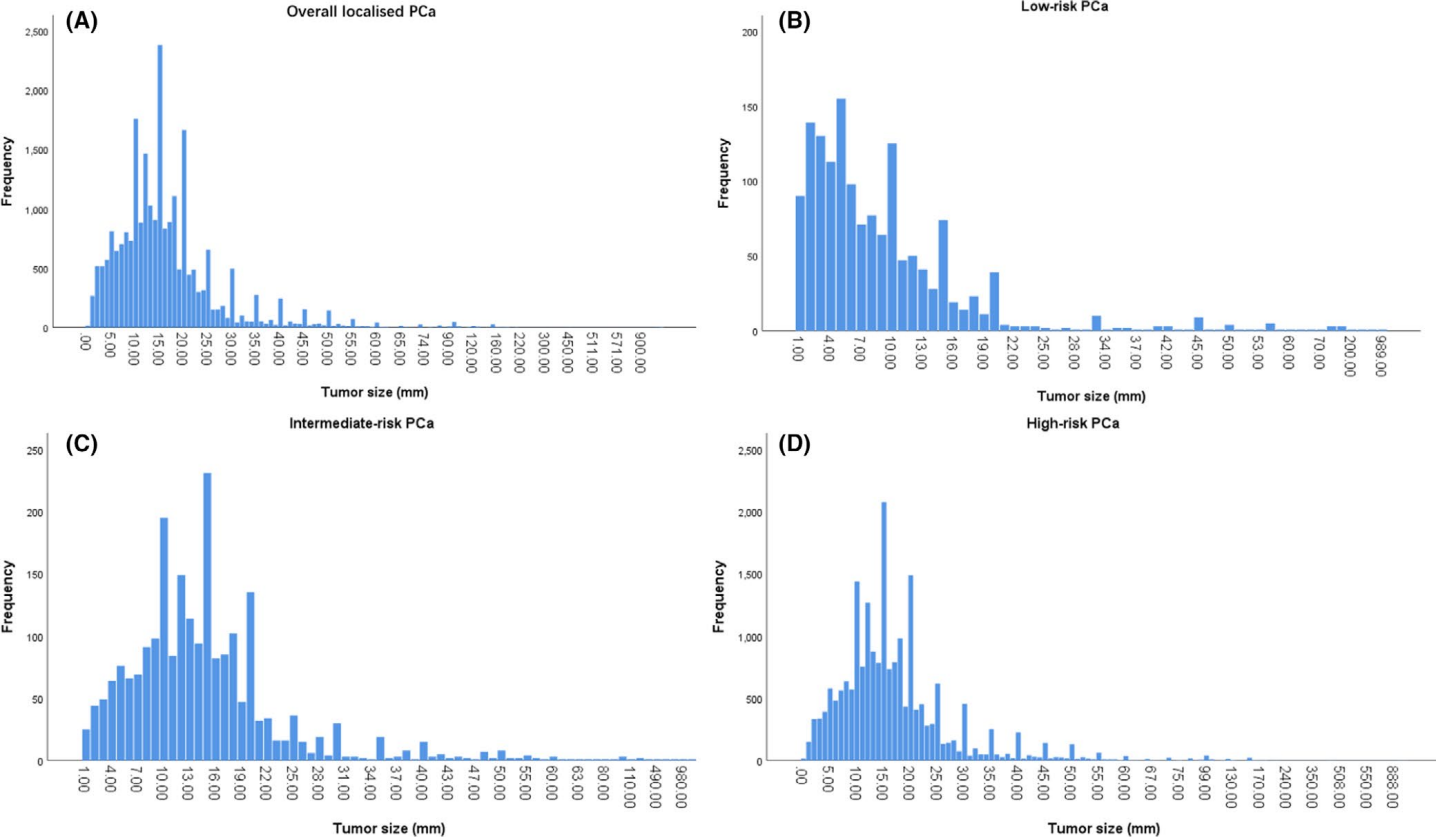
The frequency of tumor size in overall localised/low‐/intermediate‐/high‐risk prostate cancer (PCa) (A/B/C/D)

As shown in Figure 2A, the tumor size tended to be concentrated between 5 and 20 mm, which can be seen in both Figure 2C and D. According to Figure 2B, however, the frequency decreased with increasing tumor size in low‐risk PCa. Therefore, patients with localized PCa were categorized into subgroups in accordance with tumor size ≤5, 6–10, 11–15, 16–20, and ≥21 mm.

### 3.2 General characteristics

Table 1 depicts the characteristics of 24,127 patients. Among the overall localized PCa patients, 15,376 (63.5%) patients were younger than 65 years old. In addition, the white race (80.5%), married patients (76.4%), non‐paired site patients (99.7%), those with poorly differentiated tumors (51.3%), and tumor size 11–15 mm (28.1%) made up the majority of localized PCa. Moreover, most patients suffered high‐risk PCa (20,450), while few were low‐risk PCa (1491). The low‐risk patients' characteristics were similar to the overall population, except for the highest incidence of tumor grade (moderately differentiated, grade II, 72.0%) and tumor size (≤5 mm, 42.1%). In intermediate‐risk and high‐risk PCa, those patient characteristics were identical to overall localized PCa, but there was no intergroup difference for marital status groups (*p* = 0.530) and laterality groups (*p* = 0.238) in intermediate‐risk PCa, as well as no intergroup difference for marital status (*p* = 0.067) in high‐risk PCa.

**TABLE 1:** Baseline demographic and tumor characteristics of patients between different localized PCa

Variables	Localized PCa										
	Overall, *N* (%)	*p*	Low‐risk, *N* (%)		P	Intermediate‐risk, *N* (%)		*p*	High‐risk, *N* (%)		*p*
			Yes	No		Yes	No		Yes	No	
Age		<0.001			<0.001			0.314			0.004
<65	15,376 (63.5)		1050 (70.4)	14,326 (63.3)		1400 (62.8)	13,976 (63.8)		12,926 (63.3)	2450 (65.8)	
≥65	8751 (36.5)		441 (29.6)	8310 (36.7)		831 (37.2)	7920 (36.2)		7479 (36.7)	1272 (34.2)	
Race		<0.001			<0.001			<0.001			<0.001
White	19,475 (80.5)		1257 (84.3)	18,218 (80.5)		1826 (81.8)	17,649 (80.6)		16,392 (80.3)	3083 (82.8)	
Black^a^	2765 (11.8)		109 (7.3)	2568 (11.7)		179 (8.0)	2586 (11.8)		2477 (12.1)	288 (7.7)	
Other^b^	1672 (6.8)		108 (7.2)	1564 (6.9)		207 (9.3)	1465 (6.7)		1357 (6.7)	315 (8.5)	
Unknown	215 (0.9)		17 (1.1)	198 (0.9)		19 (0.9)	196 (0.9)		179 (0.9)	36 (1.0)	
Marital status		0.009			0.002			0.530			0.067
Married	18,460 (76.4)		1193 (80.0)	17,267 (76.3)		1710 (76.6)	16,750 (76.5)		15,557 (76.2)	2903 (78.0)	
Non‐married^c^	4180 (17.5)		209 (14.0)	3971 (17.5)		395 (17.7)	3785 (17.3)		3576 (17.5)	604 (16.2)	
Unknown	1487 (6.2)		89 (6.0)	1398 (6.2)		126 (5.6)	1361 (6.2)		1272 (6.2)	215 (5.8)	
Laterality		<0.001			0.001			0.238			<0.001
Non‐paired site^d^	24,040 (99.7)		1482 (99.4)	22,558 (99.7)		2221 (99.6)	21,819 (99.6)		20,337 (99.7)	3703 (99.5)	
Left	15 (0.1)		2 (0.1)	13 (0.1)		3 (0.1)	12 (0.1)		10 (0.0)	5 (0.1)	
Right	33 (0.1)		7 (0.5)	26 (0.1)		5 (0.2)	28 (0.1)		21 (0.1)	12 (0.3)	
Paired site	39 (0.2)		0	39 (0.2)		2 (0.1)	37 (0.2)		37 (0.2)	2 (0.1)	
Grade		<0.001			<0.001			<0.001			<0.001
Well, I	1492 (5.7)		277 (18.6)	1215 (5.4)		62 (2.8)	1430 (6.5)		1153 (5.7)	339 (9.1)	
Moderately, II	10,399 (42.0)		1074 (72.0)	9325 (41.2)		832 (37.3)	9567 (43.7)		8493 (41.6)	1906 (51.2)	
Poorly, III	12,013 (51.3)		128 (8.6)	11,885 (52.5)		1313 (58.9)	10,700 (48.9)		10,572 (51.8)	1441 (38.7)	
Undifferentiated, IV	19 (0.1)		0 (0.0)	19 (0.1)		1 (0.0)	18 (0.1)		18 (0.1)	1 (0.0)	
Unknown	204 (0.8)		12 (0.8)	192 (0.8)		23 (1.0)	181 (0.8)		169 (0.8)	35 (0.9)	
Tumor size		<0.001			<0.001			<0.001			<0.001
≤5 mm	2689 (9.8)		627 (42.1)	2062 (9.1)		258 (11.6)	2431 (11.1)		1804 (8.8)	885 (23.8)	
6–10 mm	4638 (18.6)		435 (29.2)	4203 (18.6)		519 (23.3)	4119 (18.8)		3684 (18.1)	954 (25.6)	
11–15 mm	6666 (28.1)		240 (16.1)	6426 (28.4)		672 (30.1)	5994 (27.4)		5754 (28.2)	912 (24.5)	
16–20 mm	4980 (21.3)		106 (7.1)	4874 (21.5)		451 (20.2)	4529 (20.7)		4423 (21.7)	557 (15.0)	
≥21 mm	5154 (22.3)		83 (5.6)	5071 (22.4)		331 (14.8)	4823 (22.0)		4740 (23.2)	414 (11.1)	

Abbreviation: PCa, prostate cancer.

^a^ Black or African American.

^b^ Includes American Indian/Alaska Native, Asian, and Asian/Pacific Islander.

^c^ Includes widowed, never married, divorced, separated, unmarried, and domestic partner.

^d^ Unilaterally, but no information concerning specific laterality.

### 3.3 Influence of different tumor size on different localized PCa stages

As we can see in Table 2, univariate logistic regression analysis of six variables was used between groups based on yes or no to a certain risk stage PCa. Final multivariate logistic regression analysis model would analyze variables with *p* < 0.05 in univariate analysis (Table 3).

**TABLE 2:** Univariate logistic regression analysis evaluating the influence of tumor size on different localized prostate cancers

Variables	Low‐risk PCa	*p*	Intermediate‐risk PCa	*p*	High‐risk PCa	*p*
	OR (95% CI)		OR (95% CI)		OR (95% CI)	
Age		<0.001		0.314		0.004
<65	Reference		Reference		Reference	
≥65	1.381 (1.232–1.549)	<0.001	0.955 (0.872–1.045)	0.314	0.897 (0.834–0.956)	0.004
Race		<0.001		<0.001		<0.001
White	Reference		Reference		Reference	
Black^a^	1.681 (1.377–2.053)	<0.001	1.495 (1.275–1.752)	<0.001	0.618 (0.544–0.703)	<0.001
Other^b^	0.999 (0.815–1.224)	0.994	0.732 (0.628–0.854)	<0.001	1.234 (1.085–1.403)	0.001
Unknown	0.804 (0.488–1.323)	0.390	1.067 (0.665–1.713)	0.787	1.069 (0.746–1.533)	0.715
Marital status		0.002		0.530		0.068
Married	Reference		Reference		Reference	
Non‐married^c^	1.313 (1.129–1.527)	<0.001	0.978 (0.872–1.097)	0.708	0.905 (0.823–0.995)	0.040
Unknown	1.085 (0.869–1.355)	0.470	1.103 (0.913–1.332)	0.311	0.906 (0.780–1.052)	0.196
Laterality		0.007		0.258		0.001
Non‐paired site^d^	Reference		Reference		Reference	
Left	0.427 (0.096–1.894)	0.263	0.407 (0.115–1.444)	0.164	2.746 (0.938–8.038)	0.065
Right	0.244 (0.106–0.563)	0.001	0.570 (0.220–1.478)	0.247	3.138 (1.543–6.384)	0.002
Paired site	/^e^	0.998	1.883 (0.454–7.818)	0.384	0.297 (0.072–1.232)	0.094
Grade		<0.001		<0.001		<0.001
Well, I	Reference		Reference		Reference	
Moderately, II	1.979 (1.712–2.288)	<0.001	0.499 (0.383–0.649)	<0.001	0.763 (0.670–0.870)	<0.001
Poorly, III	21.169 (17.028–26.315)	<0.001	0.353–0.272–0.459)	<0.001	0.464 (0.406–0.530)	<0.001
Undifferentiated,IV	/		0.780 (0.103–5.940)	0.811	0.189 (0.025–1.421)	0.105
Unknown	3.648 (2.007–6.631)	<0.001	0.341 (0.206–0.564)	<0.001	0.704 (0.480–1.034)	0.073
Tumor size		<0.001		<0.001		
≤5 mm	Reference		Reference		Reference	<0.001
6–10 mm	2.938 (2.572–3.356)	<0.001	0.842 (0.720–0.986)	0.033	0.528 (0.474–0.588)	<0.001
11–15 mm	8.142 (6.960–9.524)	<0.001	0.947 (0.814–1.101)	0.477	0.323 (0.290–0.359)	<0.001
16–20 mm	13.982 (11.309–17.286)	<0.001	1.066 (0.908–1.252)	0.437	0.257 (0.228–0.289)	<0.001
≥21 mm	18.578 (14.693–23.489)	<0.001	1.546 (1.305–1.833)	<0.001	0.178 (0.157–0.202)	<0.001

Abbreviations: CI, confidence interval; OR, odds ratio; PCa, prostate cancer.

^a^ Black or African American.

^b^ Includes American Indian/Alaska Native, Asian, and Asian/Pacific Islander.

^c^ Includes widowed, never married, divorced, separated, unmarried, and domestic partner.

^d^ Unilaterally, but no information concerning specific laterality.

^e^ Invalid value.

**TABLE 3:** Multivariate logistic regression analysis evaluating the influence of tumor size on different localized prostate cancers

Variables	Low‐risk PCa	*p*	Intermediate‐risk PCa	*p*	High‐risk PCa	*p*
	OR (95% CI)		OR (95% CI)		OR (95% CI)	
Age		<0.001		NI		0.001
<65	Reference				Reference	
≥65	1.357 (1.201–1.534)	<0.001			0.883 (0.819–0.953)	0.001
Race		<0.001		<0.001		<0.001
White	Reference		Reference		Reference	
Black^a^	1.551 (1.257–1.913)	<0.001	1.487 (1.267–1.744)	<0.001	0.631 (0.554–0.719)	<0.001
Other^b^	0.912 (0.733–1.136)	0.413	0.723 (0.619–0.843)	<0.001	1.310 (1.148–1.496)	<0.001
Unknown	0.845 (0.491–1.453)	0.542	0.988 (0.620–1.606)	0.992	1.077 (0.743–1.560)	0.696
Marital status		0.008		NI		NI
Married	Reference					
Non‐married^c^	0.991 (0.781–1.256)	0.991				
Unknown	1.276 (0.971–1.675)	0.080				
Laterality		0.003		NI		0.002
Non‐paired site^d^	Reference				Reference	
Left	0.249 (0.050–1.227)	0.263			2.835 (0.939–8.556)	0.064
Right	0.196 (0.074–0.515)	0.001			3.068 (1.468–6.413)	0.003
Paired site	/^e^	0.997			0.301 (0.071–1.279)	0.104
Grade		<0.001		<0.001		<0.001
Well, I	Reference		Reference		Reference	
Moderately, II	1.480 (1.269–1.726)	<0.001	0.465 (0.357–0.607)	<0.001	0.940 (0.821–1.076)	0.370
Poorly, III	11.721 (9.353–14.689)	<0.001	0.314 (0.241–0.409)	<0.001	0.685 (0.596–0.788)	<0.001
Undifferentiated,IV	/	0.998	0.692 (0.091–5.281)	0.722	0.292 (0.038–2.223)	0.234
Unknown	2.927 (1.582–5.417)	<0.001		<0.001	0.854 (0.576–1.265)	0.431
Tumor size		<0.001		<0.001		<0.001
≤5 mm	Reference		Reference		Reference	
6–10 mm	2.284 (1.988–2.623)	<0.001	0.946 (0.806–1.109)	0.493	0.564 (0.506–0.629)	<0.001
11–15 mm	5.481 (4.662–6.443)	<0.001	1.120 (0.960–1.308)	0.150	0.357 (0.320–0.398)	<0.001
16–20 mm	8.686 (6.992–10.791)	<0.001	1.297 (1.099–1.529)	0.002	0.288 (0.255–0.325)	<0.001
≥21 mm	11.940 (9.404–15.161)	<0.001	1.887 (1.586–2.245)	<0.001	0.197 (0.173–0.225)	<0.001

Abbreviations: CI, confidence interval; NI, not included in the multivariate survival analysis; OR, odds ratio; PCa, prostate cancer.

^a^ Black or African American.

^b^ Includes American Indian/Alaska Native, Asian, and Asian/Pacific Islander.

^c^ Includes widowed, never married, divorced, separated, unmarried, and domestic partner.

^d^ Unilaterally, but no information concerning specific laterality.

^e^ Invalid value.

When predicting low‐risk PCa, univariate and multivariate logistic regression analyses indicated that age, race, marital status, laterality, grade, and tumor sizes were independent predictors (Tables 2 and 3). When comparing to tumor size ≤5 mm, there was a higher probability of low‐risk PCa in patients who had tumor size 6–10, 11–15, 16–20, and ≥21 mm (All *p* < 0.001, Table 3). For intermediate‐risk PCa, the results of univariate and multivariate logistic regression analysis indicated that race, grade, and tumor sizes were independent predict factor (Tables 2 and 3). When comparing to tumor size ≤5 mm, patients with tumor sizes 16–20 and ≥21 mm had a higher probability of intermediate‐risk PCa (Table 3). In high‐risk PCa, the results of univariate and multivariate logistic regression analyses determined that age, race, laterality, grade, and tumor sizes were independent predict factors (Tables 2 and 3). According to patients that had tumors ≤5 mm, patients with tumor sizes 6–10, 11–15, 16–20, and ≥21 mm had a lower risk of high‐risk PCa (All *p* < 0.001, Table 3).

### 3.4 Survival analyses according to tumor size

**FIGURE 3:**
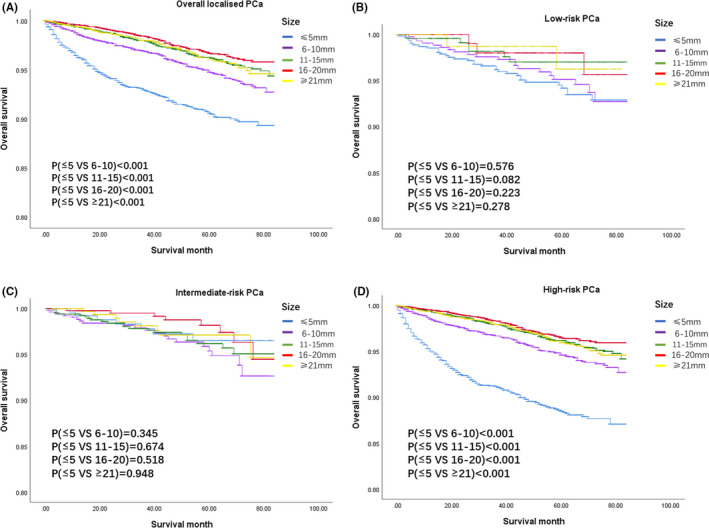
Kaplan‐Meier survival curves of overall survival to different tumor size group in overall localised/ low‐/intermediate‐/high‐risk prostate cancer (PCa) (A/B/C/D)

As is shown in Figure 3A, patients with tumor size ≤5 mm had the worst OS than others in overall patients and patients who had tumors 16–20 mm had the best OS (*p* < 0.001). However, the difference was not significant in low‐risk PCa and intermediate‐risk PCa (*p* = 0.308, Figure 3B; *p* = 0.411, Figure 3C). In high‐risk PCa, patients with tumor size ≤5 mm had the worst OS than other groups (*p* < 0.001, Figure 3D), while the groups with tumor size >10 mm had no significant difference (Figure 3D). Univariate and multivariate Cox regression analyses showed that age was an independent risk factor across all diseases (all *p* < 0.001, Tables 4 and 5). Besides, in overall localized PCa and high‐risk PCa, the pathological grade was an independent risk factor (all *p* < 0.001, Tables 4 and 5). In overall localized PCa patients with tumor size 6–10, 11–15, 16–20, and ≥21 mm (All *p* < 0.001; Table 5) had a better OS compared to those with tumor size ≤5 mm. In high‐risk PCa, patients with tumor sizes 6–10, 11–15, 16–20, and ≥21 mm All *p* < 0.001; Table 5) had a better OS compared to patients with tumor size ≤5 mm.

**TABLE 4:** Univariate cox regression analysis evaluating the influence of tumor size on different localized prostate cancers

Variables	Localized PCa							
	Overall	*p*	Low‐risk	*p*	Intermediate‐risk	*p*	High‐risk	*p*
	HR (95% CI)		HR (95% CI)		HR (95% CI)		HR (95% CI)	
Age		<0.001		<0.001		<0.001		<0.001
<65	Reference		Reference		Reference		Reference	
≥65	3.263 (2.853–3.732)	<0.001	4.297 (2.590–7.129)	<0.001	3.467 (2.069–5.810)	<0.001	3.188 (2.759–3.684)	<0.001
Race		0.144		0.137		0.726		0.108
White	Reference		Reference		Reference		Reference	
Black^a^	1.132 (0.931–1.376)	0.215	0.582 (0.182–1.857)	0.360	1.140 (0.490–2.651)	0.762	1.154 (0.941–1.416)	0.170
Other^b^	0.800 (0.601–1.065)	0.126	0.391 (0.096–1.603)	0.192	0.653 (0.236–1.805)	0.411	0.866 (0.638–1.176)	0.357
Unknown	0.587 (0.220–1.568)	0.288	3.337 (0.814–13.675)	0.094	2.008 (0.277–14.527)	0.490	0.175 (0.025–1.242)	0.081
Marital status		<0.001		0.109		0.802		<0.001
Married	Reference		Reference		Reference		Reference	
Non‐married^c^	1.792 (1.547–2.077)	<0.001	1.650 (0.896–3.038)	0.108	1.039 (0.540–2.002)	0.908	1.876 (1,604–2.194)	<0.001
Unknown	0.811 (0.594–1.109)	0.460	0.281 (0.039–2.038)	0.209	1.366 (0.544–3.432)	0.507	0.808 (0.577–1.133)	0.217
Laterality		0.881		0.913		0.988		0.474
Non‐paired site^d^	Reference		Reference		Reference	0.842	Reference	
Left	1.830 (0.258–13.007)	0.546	/	0.833	/	0.842	2.914 (0.410–20.716)	0.285
Right	1.461 (0.365–5.853)	0.592	/	0.711	/	0.795	2.280 (0.569–9.134)	0.244
Paired site	/^e^	0.901	——^f^	——	/	0.875	/	0.902
Grade		<0.001		0.269		0.816		<0.001
Well, I	Reference		Reference		Reference		Reference	
Moderately, II	0.483 (0.376–0.621)	<0.001	0.518 (0.263–1.023)	0.058	/	0.895	0.468 (0.357–0.614)	<0.001
Poorly, III	0.367 (0.285–0.471)	<0.001	0.569 (0.207–1.562)	0.274	/	0.890	0.331 (0.252–0.434)	<0.001
Undifferentiated,IV	0.469 (0.065–3.375)	0.452	1.053 (0.135–8.209)	0.960	/	1.000	0.443 (0.061–3.199)	0.420
Unknown	0.447 (0.224–0.895)	0.023	——	——	/	1.000	0.440 (0.210–0.919)	0.029
Tumor size		<0.001		0.330		0.427		<0.001
≤5 mm	Reference		Reference		Reference		Reference	
6–10 mm	0.533 (0.442–0.643)	<0.001	0.853 (0.489–1.486)	0.575	1.037 (0.376–2.861)	0.943	0.435 (0.354–0.536)	<0.001
11–15 mm	0.374 (0.310–0.451)	<0.001	0.472 (0.198–1.126)	0.091	1.571 (0.692–3.567)	0.280	0.302 (0.246–0.370)	<0.001
16–20 mm	0.305 (0.245–0.379)	<0.001	0.485 (0.149–1.581)	0.230	1.262 (0.559–2.849)	0.576	0.251 (0.198–0.316)	<0.001
≥21 mm	0.372 (0.303–0.457)	<0.001	0.464 (0.111–1.934)	0.292	0.734 (0.275–1.956)	0.536	0.303 (0.243–0.376)	<0.001

Abbreviations: CI, confidence interval; HR, hazard ratio; PCa, prostate cancer.

^a^ Black or African American.

^b^ Includes American Indian/Alaska Native, Asian, and Asian/Pacific Islander.

^c^ Includes widowed, never married, divorced, separated, unmarried, and domestic partner.

^d^ Unilaterally, but no information concerning specific laterality.

^e^ Invalid value.

^f^ Countless value.

**TABLE 5:** Multivariate cox regression analysis evaluating the influence of tumor size on different localized prostate cancers

Variables	Localized PCa							
	Overall	*p*	Low‐risk	*p*	Intermediate‐risk	*p*	High‐risk	*p*
	HR (95% CI)		HR (95% CI)		HR (95% CI)		HR (95% CI)	
Age		<0.001		<0.001		<0.001		<0.001
<65	Reference		Reference		Reference		Reference	
≥65	3.344 (2.923–3.827)	<0.001	3.263 (2.853–3.732)	<0.001	3.467 (2.069–5.810)	<0.001	3.336 (2.885–3.858)	<0.001
Race		NI		NI	NI			NI
White								
Black^a^								
Other^b^								
Unknown								
Marital status		NI		NI		NI		NI
Married								
Non‐married^c^								
Unknown								
Laterality		NI		NI		NI		NI
Non‐paired site^d^								
Left								
Right								
Paired site								
Grade		<0.001		NI		NI		<0.001
Well, I	Reference						Reference	
Moderately, II	0.521 (0.405–0.671)	<0.001					0.524 (0.398–0.689)	<0.001
Poorly, III	0.441 (0.340–0.570)	<0.001					0.412 (0.311–0.545)	<0.001
Undifferentiated,IV	0.554 (0.077–3.992)	0.558					0.589 (0.082–4.260)	0.600
Unknown	0.504 (0.252–1.009)	0.053					0.556 (0.266–1.165)	0.120
Tumor size		<0.001		NI		NI		<0.001
≤5 mm	Reference						Reference	
6–10 mm	0.580 (0.479–0.701)	<0.001					0.494 (0.401–0.610)	<0.001
11–15 mm	0.422 (0.347–0.512)	<0.001					0.352 (0.285–0.434)	<0.001
16–20 mm	0.343 (0.273–0.430)	<0.001					0.295 (0.232–0.376)	<0.001
≥21 mm	0.412 (0.334–0.510)	<0.001					0.348 (0.278–0.436)	<0.001

Abbreviations: CI, confidence interval; HR, hazard ratio; NI, not included in the multivariate survival analysis; PCa, prostate cancer.

^a^ Black or African American.

^b^ Includes American Indian/Alaska Native, Asian, and Asian/Pacific Islander.

^c^ Includes widowed, never married, divorced, separated, unmarried, and domestic partner.

^d^ Unilaterally, but no information concerning specific laterality.

## 4 DISCUSSION

In this study, the first time we tried to figure out the role of tumor size in predicting high‐risk diseases in localized PCa. We found that tumor size ≤5 mm was significantly associated with high‐risk PCa and patients with tumor size ≤5 mm had a poorer prognosis.

Localized PCa is defined as a stage cT1‐2c tumor in most guidelines.^4^, ^9^ But the NCCN guideline described that PCa at any T‐stage could be defined as localized PCa, as long as there is no lymph node involvement (N0) or metastases (M0).^10^ The guidelines of Cancer Control Alberta and the SIU define cT1–cT3 as localized PCa with the exception of cT4.^9^, ^11^ In this study, we considered that most guidelines agree on the role of risk‐stratification protocol for localized PCa as a tool to speculate prognosis and to provide adjunctive information for choosing the appropriate treatment modalities.^4^, ^5^, ^12^ Different thresholds were used to identify the different risk groups. Low‐, intermediate‐, and high‐risk groups are commonly used, usually in combination with TNM stage, PSA level, and Gleason score.^13^, ^14^

Tumor size, as the most direct manifestation of cancer, has always been the focus of disease stratification and prognosis. The study showed that tumor size was positively correlated with low‐, intermediate‐risk PCa. Among the low‐risk PCa, most cases had tumor size ≤5 mm and only 5.6% of patients had tumor size ≥21 mm. It seems to mean that tumor size ≤5 mm was more likely to develop low‐risk PCa. However, logistic regression analysis showed that tumor sizes ≥21 mm was significantly predictive of low‐risk PCa and patients with tumor size ≥21 mm were 11.9 times than patients with tumor size ≤5 mm. Similarly, when comparing to those with tumor size ≤5 mm, patients with tumor size ≥21 mm were 1.9 times to suffer intermediate‐risk PCa. Because there are few studies on the value of tumor size in the diagnosis, stratification, and prognosis of PCa, we do not have many findings of other studies as a control reference. For patients with tumor size ≥21 mm and without the invasion of prostate capsule, lymph node involvement (N0) or metastases (M0), serial digital rectal examination (at least once yearly), PSA (at least once, every 6 months), and repeated biopsy (at a minimum interval of 3–5 years) of joint inspection is very necessary.^15^ Once tumor is found to progressively increase in diameter, its potential for progression should be considered and treatment strategies, such as RP, should be operated.

In our study, tumor size ≤5 mm was significantly associated with high‐risk PCa. Subsequently, we found that patients with tumor size ≤5 mm had the worst OS. The result was consistent with our prediction that we got from OR, however, seems to run counter to common sense. We suspected that this might result from that tumor size ≤5 mm may not benefit as much from surgery as others. Besides, short PSA doubling time of those patients may play an important role in poor prognosis.^16^ Some scholars believe that 10 mm is the critical value for the selection of treatment, and when less than 10 mm, active surveillance can be adopted.^17^, ^18^ Due to the limitations of modern imaging, small tumors are sometimes difficult to detect. Besides, as more and more people realized the seriousness of overdiagnosis and overtreatment, high‐risk PCa with tumor size ≤5 mm is more difficult to diagnose. The latest research suggested that MRI‐targeted biopsies (MRI‐TBx) can maximize the identification of tumors smaller than 6 mm.^19^ Once the diagnosis is made, RP is a reasonable choice for selected patients with low tumor volume. Besides, enlarged pelvic lymph node dissection is recommended for all high‐risk PCa.^20^ Moreover, using external‐beam radiation therapy (EBRT) with 76–78 Gy in combination with long‐term androgen deprivation therapy (2–3 years) is effective for high‐risk PCa patients.^21^ In conclusion, for tumors size ≤5 mm, RP in combination with other therapeutic measures may improve the prognosis than RP alone.

There are several limitations. First, tumor size ≤5 mm had the worst prognosis, but in Cox regression analysis, we found that HR value began to rebound after reaching the minimum in the 16–20 mm tumor size group. Therefore, there might exist a critical value of tumor size influencing the prognosis. Due to the characteristics of the selected samples, this stratification has not been made yet, but it can be predicted that the prognosis will worsen when the tumor size reaches a certain value, which needs to be confirmed by subsequent studies. Moreover, since the tumor size in this study was obtained by postoperative pathology, all the samples were selected after RP surgery. The results would become more convincing if validated by the imaging data. It has been reported that contrast‐enhanced transrectal ultrasonography is valuable in the measurement of the size of PCa, especially for those with a diameter >10 mm.^22^ This provides us with a prospective research idea in clinical practice, which means predicted by imaging examination and then confirmed by pathological examination.

In conclusion, this study showed that tumor sizes ≥21 mm were an independent predictor for low‐, intermediate‐risk PCa. However, we should be on high alert for tumor size ≤5 mm or even negative on imaging tests due to its significant association with high‐risk PCa.

## CONFLICT OF INTEREST

The authors report no conflicts of interest in this work.

## AUTHOR CONTRIBUTIONS

All authors contributed toward data analysis, drafting, and writing the paper, gave final approval of the version to be submitted, and agree to be accountable for all aspects of the work.

## ETHICAL APPROVAL

For the institutional cohorts, data were extracted from the Surveillance, Epidemiology, and End Results database. This article does not contain any studies with human participants performed by any of the authors.

## Data Availability

The dataset analyzed during the current study is available in the Surveillance, Epidemiology, and End Results (SEER) database and can be accessed in detail through the utilization of SEER*Stat (https://seer.cancer.gov/data/).
